# Mechanisms of scramblases in regulating hepatic lipoprotein secretion and autophagy

**DOI:** 10.1016/j.jlr.2026.100979

**Published:** 2026-01-13

**Authors:** Allen Chen, Chen Zhang, Wen-Xing Ding, Hong-Min Ni

**Affiliations:** 1 Department of Pharmacology, Toxicology and Therapeutics, University of Kansas Medical Center, Kansas City, Kansas, USA; 2 Department of Internal Medicine, University of Kansas Medical Center, Kansas City, Kansas, USA

**Keywords:** autophagy, endoplasmic reticulum, liver injury, MASLD, scramblases, steatosis, VLDL

## Abstract

Lipoprotein secretion is a complex, highly regulated multi-step process that ensures the efficient transport of lipids from cells into the bloodstream, supporting overall metabolic health. The secretion of very-low-density lipoprotein (VLDL) relies on the proper assembly and movement of phospholipids within cellular membranes, particularly the endoplasmic reticulum (ER). Changes in the composition and dynamics of phospholipids can affect lipoprotein size, lipid loading, and, ultimately, the ability of VLDL to be secreted. Scramblases are a class of transmembrane proteins that facilitate the movement of phospholipids between the inner and outer leaflets of membrane bilayers in a bidirectional, energy-independent manner, serving an equilibrating function. They help balance phospholipids to reduce mechanical strain and curvature in membranes, which is critical for various cellular processes, including autophagy. Recent evidence suggests that the scramblases TMEM41B and VMP1 play essential roles in regulating VLDL secretion. Loss of hepatic TMEM41B or VMP1 results in severe defects in VLDL secretion and leads to the rapid development of metabolic dysfunction-associated steatohepatitis (MASH) in mice. In this review, we discuss the latest advancements in understanding these lipid scramblases, highlighting their similarities and distinct roles in maintaining lipid homeostasis, membrane dynamics, autophagy, and VLDL secretion in the pathogenesis of MASH.

Metabolic dysfunction-associated steatotic liver disease (MASLD), formerly known as non-alcoholic fatty liver disease, is characterized by more than five percent hepatic steatosis along with at least one cardiometabolic risk factor, such as elevated body mass index (BMI), insulin resistance, hypertension, or dyslipidemia, and no history of excessive alcohol use (1, 2, 3). MASLD has become one of the leading causes of chronic liver disease globally, and it was shown that 38% of all adults and 7%–14% of children and adolescents have MASLD around the world (4). Though MASLD is generally asymptomatic, left untreated, the disease can progress to metabolic dysfunction-associated steatohepatitis (MASH) in which lipid overload precipitates cell death and inflammation. Though cardiovascular disease remains the most common cause of mortality among individuals with MASLD/MASH, chronic liver diseases, including MASH, can eventually progress to more severe end-stage conditions, including cirrhosis and hepatocellular carcinoma (HCC) (3, 5).

High prevalence of MASLD, coupled with the rising incidence of other metabolic diseases such as obesity and type 2 diabetes mellitus (T2DM), indicates that MASLD and sequelae may soon become a significant burden to the population worldwide. Currently, there are few pharmacological treatments available for MASLD or MASH, with resmetirom being the first FDA-approved drug that targets MASH explicitly through its hepatic thyroid hormone receptor (THR) agonist activity (6). More recently, the FDA has approved semaglutide, a GLP-1 receptor agonist found in the weight-loss drugs Wegovy and Ozempic, for the treatment of MASH patients. It showed improved histological markers of fibrosis and inflammation and reduced the hepatic expression of fibrosis-related and inflammation-related gene pathways in two preclinical MASH models (7, 8). Additionally, survodutide, a dual glucagon/GLP-1 receptor agonist, and retatrutide, a triple receptor agonist targeting gastric inhibitory polypeptide (GIP), GLP-1, and glucagon receptors, are currently in clinical trials that could provide new options for this serious chronic liver disease. However, any therapeutic effects on MASLD/MASH are probably secondary to systemic metabolic improvement.

Excessive hepatic lipid accumulation leading to MASLD and MASH can be considered a consequence of a breakdown in the balance between lipid accumulation and elimination due to dysfunctional control over the two major mechanisms of hepatic lipid accumulation, hepatic lipid uptake and de novo lipogenesis, and the two major mechanisms of hepatic lipid loss, fatty acid oxidation and secretion of hepatic triglyceride via very low density lipoprotein (VLDL) (Fig. 1A).

**Fig. 1:**
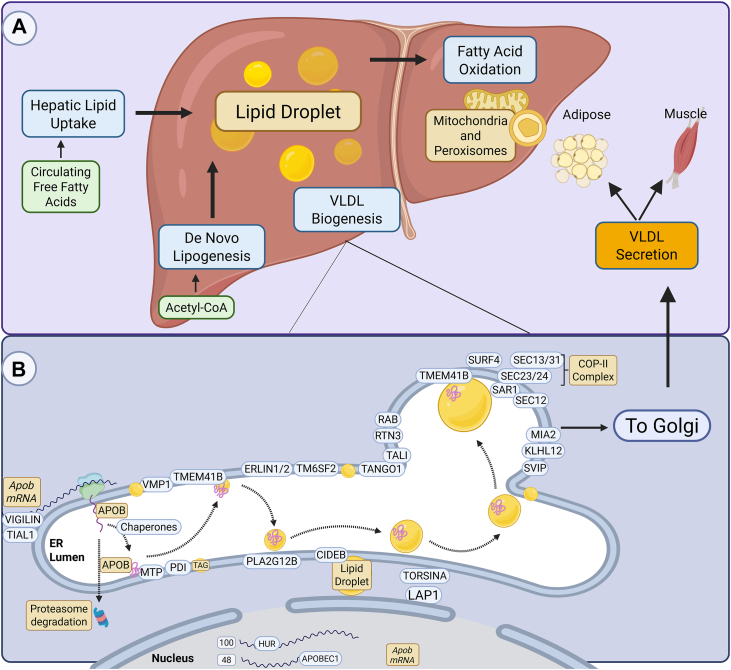
A schematic of hepatic processes contributing to lipid homeostasis and key players in regulating VLDL secretion. A: A scheme of hepatic processes regulating lipid homeostasis. Hepatic lipid homeostasis is maintained through the interplay of four processes, de novo lipogenesis and lipid uptake from circulation which increase hepatic lipid content, and VLDL secretion and fatty acid oxidation, which decrease hepatic lipid content. Hepatic lipid uptake from circulation includes FFA originating from adipose tissue and lipoproteins from the bloodstream. De novo lipogenesis uses Acetyl-CoA from the Krebs Cycle to produce palmitate, and subsequently, triglycerides are synthesized by esterifying fatty acids to glycerol and stored in lipid droplets (LD). Fatty acid oxidation occurs within mitochondria and peroxisomes to break down Lipids for energy. Triglycerides and cholesterol can also be packed into VLDL and secreted to the circulation to deliver lipids to other tissues such as adipose and muscle. B: A proposed scheme for the key players regulating cellular VLDL biogenesis and secretion. One of the key steps in the biogenesis of VLDL begins with the transcription and translation regulation of APOB. The mRNA of *APOB* can be regulated by RNA binding protein human antigen R (HUR), TIA1 cytotoxic granule-associated RNA-binding protein like 1 (TIAL1), and VIGILIN. *APOB* mRNA can also be edited by APOBEC1 in the nucleus, which generates *APOB48* mRNA, encoding a truncated (48%) portion of the full-length APOB100 protein in mouse but not in human hepatocytes. APOB is translated and enters the ER lumen via chaperones, where PDI and MTP, which form a heterodimeric complex, bind to and facilitate the lipidation of APOB. Misfolded APOB or insufficiently lipidated APOB is ubiquitinylated and degraded by the proteasome. The two lipid scramblases VMP1 and TMEM41B then facilitate the budding off of VLDL from the ER membrane into the ER lumen. The nascent VLDL is lipid-poor and becomes more mature and well-lipidated through several mechanisms. These include the AAA + ATPase TORSINA, which is activated by lamina-associated polypeptide 1 (LAP1) at the nuclear envelope; phospholipase A2 group XIIB (PLA2G12B); ER lipid raft proteins 1 and 2 (ERLIN 1 and 2); and transmembrane 6 superfamily member 2 (TM6SF2). Additionally, CIDEB aids more lipids to the VLDL from cytosolic LDs. Next, VLDL exits ER lumen and trafficking to the Golgi, a process regulated by multiple proteins such as transport and Golgi organization protein 1 (TANGO1), TANGO1-like (TALI), Kelch-like protein 12 (KLHL12), small valosin-containing protein-interacting protein (SVIP), reticulon 3 (RTN3), several small Rab GTPases (RAB), and surfeit locus protein 4 (SURF4). When the VLDL particle encounters the ER exit site, it forms a ternary complex with TMEM41B and SURF4. COPII vesicle formation is mediated by the activation of the GTPase secretion-associated RAS-related GTPase 1B (SAR1B). This GTPase recruits SEC23 and SEC24 as the inner coat proteins and SEC13 and SEC31 as the outer coat proteins. These proteins facilitate the transport of VLDL to the Golgi complex, where additional or final lipidation of VLDL occurs before it is secreted out of hepatocytes.

## Focus of this review

The contributions and mechanisms of lipid metabolism (lipogenesis, uptake, and beta-oxidation) in the development of MASLD/MASH have been extensively reviewed recently (9, 10). The mechanisms of lipoprotein biogenesis and secretion have also been elegantly reviewed lately (11, 12). In this review, we focus on discussing the current understanding of how emerging lipid scramblases contribute to hepatic lipoprotein biogenesis and secretion, as well as their role in autophagy during the pathogenesis of MASLD/MASH. We will briefly review the current understanding of VLDL biogenesis and the secretion machinery, as some of these may be directly or indirectly affected by lipid scramblases.

## VLDL biogenesis, intracellular trafficking, and secretion

Lipoproteins are a diverse group of lipid-protein particles of various sizes and compositions, typically consisting of a core of hydrophobic triglyceride and cholesterol ester (CE) surrounded by a surface layer of hydrophilic apolipoproteins, free cholesterol, and a monolayer of amphipathic phospholipids (13). The main function of lipoproteins is to transport hydrophobic lipids derived from dietary and endogenous fat to peripheral tissues via the circulatory system for energy utilization or storage (13). In addition to facilitating lipid transport, structural apolipoproteins play crucial roles in cell signaling by serving as ligands for cell surface receptors that initiate cellular responses. These responses include the clearance of lipoproteins and the activation of inflammatory pathways. For instance, APOE functions as a ligand for the LDL receptor, which facilitates the uptake of lipoproteins by cells. Similarly, APOM can bind sphingosine-1-phosphate (S1P) to HDL, forming a complex that activates signaling pathways to maintain vascular integrity and reduce inflammation (14).

In the liver, hepatic lipids are too hydrophobic to circulate freely and must be packaged in lipoprotein VLDL or transformed into more water-soluble forms. VLDL is initially assembled through co-translational lipidation within hepatocytes around the structural APOB100 protein, an N-terminal amino acid domain consisting of about 1,000 amino acids (15). APOB exists in two forms: the full-length version, APOB100, and the truncated version, APOB48. APOB48 is produced through editing of *APOB* mRNA, resulting in a shorter protein that contains the N-terminal 48% of the full-length protein. In humans, the RNA-editing enzyme APOBEC1 is only expressed in the gut, leading to the production of chylomicrons, a type of lipoprotein in the small intestine that uses APOB48 to transport dietary triglycerides, while hepatic VLDL uses APOB100. In contrast, in mice, APOBEC1 is also present in the liver, enabling the formation of VLDL that contains APOB48. Several RNA-binding proteins, including Vigilin (16), TIA1 cytotoxic granule-associated RNA binding protein-like 1 (TIAL1) (17), and human antigen R (HUR) (18), have been shown to play critical roles in regulating APOB expression. Furthermore, the successful translation and folding of functional APOB100 depend on the activity of multiple chaperone proteins. Insufficient chaperone activity, combined with a lack of lipidation or improper APOB translocation by microsomal triglyceride transfer protein (MTP), results in ubiquitination and degradation of the synthesized APOB polypeptide (19). Indeed, co-translational lipidation, which is necessary to stabilize APOB so it can be secreted from the hepatocyte as VLDL, relies on the ER-resident protein MTP, which forms a heterodimeric complex with protein disulfide isomerase (PDI) (Wetterau, Combs *et al.* 1991), and stabilizes APOB by shuttling phospholipids and CE within the ER membrane to the lipid-binding pocket of APOB (20). The lipidated APOB then buds off from the inner leaflet of the ER membrane into the ER lumen, which requires the two recently discovered phospholipid scramblases vacuole membrane protein 1 (VMP1) and transmembrane protein 41B (TMEM41B) (21, 22, 23), which we will discuss in greater detail later in the phospholipid scramblases section.

Although the exact mechanisms are not fully understood, after the initial lipidation, mainly mediated by MTP, more bulk triglycerides can be transferred to the APOB lipoprotein to form mature VLDL from the ER lumenal lipid droplets via Phospholipase A2 group 12B (PLA2G12B) in the ER lumen (24). In addition, ER lipid raft protein 1 (ERLIN1) and ERLIN2 and the transmembrane 6 superfamily member 2 (TM6SF2) form a complex to stabilize APOB (25). Moreover, the inner nuclear membrane protein lamina-associated polypeptide 1 (LAP1) binds to and activates torsinA, an AAA + ATPase that also regulates APOB lipidation and stabilization through less clear mechanisms (26). Additionally, triglycerides can be transferred from cytosolic LD to the APOB molecule via triglyceride lipolysis or direct transfer by the protein cell death-inducing DFFA-like effector B (CIDEB) (27). After sufficient lipidation of the APOB protein, the nascent VLDL may exit the luminal side of the ER membrane to be transported to the ER exit site for delivery to the Golgi apparatus. This process involves multiple protein complexes, including TANGO1, TALI, the cargo receptor SURF4, the small GTPase secretion-associated RAS-related protein 1B (SAR1B), and likely TMEM41B, as well as the COP-II complex, as discussed in detail below (21, 22, 23, 28).

Assembly of the COP-II complex begins with the activation of cytosolic small GTPase SAR1 by SEC12, an ER-resident enzyme that functions as a guanine-nucleotide exchange factor (GEF) for SAR1 activation (29, 30). Among mammals, there are two SAR1 isoforms, SAR1A and SAR1B (31, 32), with only modest differences in activity, though some studies have suggested that SAR1B has a greater role in mediating transport of large cargo vesicles (31, 33). SAR1-GTP then recruits SEC23/SEC24 to form the inner coat complex and SEC13/SEC31 as the outer COP-II coat (34, 35). The formation of the COP-II coat complex, in addition to its role in signaling, is believed to assist in deformation of the ER membrane to generate the transport vesicle (36), with active SAR1A-GTP potentiating membrane tubulation to promote formation of the transport vesicle and vesicle scission, separating the fully assembled COP-II complex from the ER (37). SAR1-GTP stabilizes the curved, budding ER membrane by recruiting the inner coat proteins, the SEC23/SEC24 lattice, which consists of concave-shaped, positively charged proteins (34). Furthermore, SEC23 contains a guanine nucleotide activating protein (GAP) that can hydrolyze GTP on SAR1 following recruitment of the inner coat complex, promoting progression of vesicle budding (38). Only after the binding of the SEC23/SEC24 inner coat, the COP-II outer cage, consisting of SEC13 and SEC31, binds directly to SEC23, allowing recruitment (39).

Formation of the COP-II complex is important for the transport of multiple cargo types from the ER. Specific to large cargoes, such as in VLDL secretion, receptor proteins, including TANGO1 and TANGO1-like, are required to recruit VLDL to these COP-II sites (28). Other proteins implicated in facilitating COP-II complex formation in VLDL secretion include kelch-like protein 12 (KLHL12), melanoma inhibitory activity protein 2 (MIA2), SURF4, SEC22b, small VCP-interacting protein (SVIP), ApoC1, reticulon 3 (RTN3), and CIDEB (12, 23, 40, 41, 42). Although it is not entirely clear whether these proteins work together or separately in regulating the COP-II complex, loss of any of these proteins has been shown to reduce plasma lipid levels by impairing VLDL secretion.

Recent evidence suggests that COP-II complex proteins can undergo phase separation, leading to condensation through a manganese-dependent process (43). Manganese directly binds to SEC23/24, promoting its condensation and regulating lipoprotein transport. Importantly, dietary restriction of manganese has been shown to improve pathological dyslipidemia and atherosclerosis in mice (43). Furthermore, many Rab GTPases, including RAB1B, RAB23, and GP73, a Golgi-resident Rab GTPase-activating protein, have also been linked to facilitating intracellular vesicle and VLDL transport throughout the cell (44). While it has been documented that VLDL can be further lipidated at the Golgi and eventually exported from the Golgi for transport to the plasma membrane and secretion from hepatocytes (45, 46), fewer studies have focused on the Golgi process than on the process occurring in the ER. The potential roles of key molecules, including the two ER scramblase proteins VMP1 and TMEM41B (discussed in more detail below), in regulating VLDL secretion are summarized in Fig. 1B.

## Scramblase proteins

Eukaryotic cells have a plasma membrane that separates them from their environment, and internal organelle membranes create intracellular compartments that separate them from the cytosol so these organelles can perform their complex and unique roles. Both membranes are phospholipid bilayers with embedded proteins, but organelle membranes establish distinct microenvironments for functions like protein synthesis, energy production, and waste clearance. For example, the ER and Golgi Apparatus feature interconnected membranes essential for protein and lipid synthesis and modification (47). Cell and organelle membranes are dynamic and can bend into curves through membrane remodeling, which is crucial for vesicle formation, autophagy, and maintaining organelle shape.

**Fig. 2:**
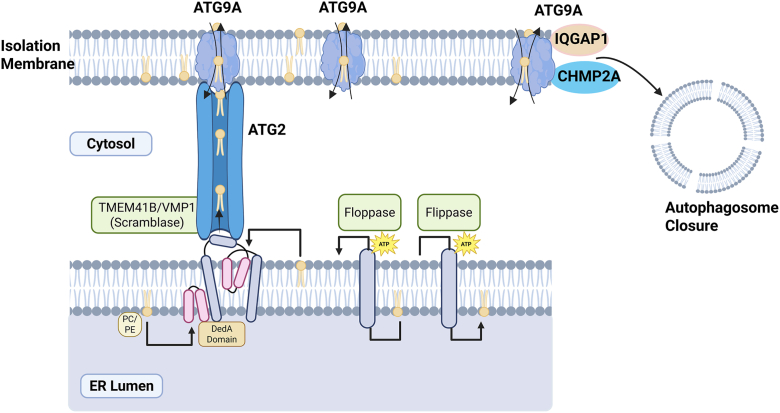
A diagram showing the function of phospholipid flipping enzymes and the role of scramblases in autophagosome formation. Most membrane-bound organelles consist of double membrane structures, sequestering the nonpolar groups of phospholipids from the aqueous cellular environment. As such, spontaneous flipping of phospholipids is rare, requiring the polar phosphate glycerol group to travel through the hydrophobic membrane compartment. Within the cell, multiple types of phospholipid-flipping proteins exist: (1) Scramblases facilitate flipping of phospholipids bi-directionally and generally serving to balance phospholipid content between the inner and outer leaflets of membrane bilayers to prevent structural damage from mismatched bilayers. (2) Floppases move phospholipids from the cytosolic compartment to the luminal or extracellular leaflet, requiring ATP to move phospholipids up their concentration gradient. (3) Flippases, perform the opposite, using ATP to catalyze the transport of lipids from the luminal or extracellular leaflet to the cytosolic leaflet of membrane bilayers. TMEM41B and VMP1 are ER-resident membrane scramblases that share an evolutionarily conserved domain called the VTT (VMP1, TMEM41B, and Tvp38) domain. This domain is homologous to regions found in many bacterial DedA family proteins and is part of the DedA superfamily. The DedA domain features two reentrant loops facing each other within the membrane and may function as an ion-coupled transporter and possess lipid-binding capabilities. VMP1 and TMEM41B may regulate the balance of phospholipids (such as phosphatidylcholine and phosphatidylethanolamine) between the inner and outer leaflets of the ER lipid bilayer and donate phospholipids to the isolation membrane via the lipid transporter protein ATG2. On the isolation membrane, another scramblase, ATG9A, further balances the phospholipid distribution between the inner and outer leaflets to facilitate isolation membrane elongation, and it cooperates with IQGAP1 and CHMP2A for the closure of autophagosomes.

One rarely discussed type of membrane protein involved in cellular membrane remodeling and autophagy is the phospholipid scramblase, which has only recently gained attention in research. Phospholipid scramblases are mainly known for their role in autophagosome and lipid droplet (LD) formation, mainly by balancing the number of phospholipids between the two leaflets of phospholipid bilayers (48). Unlike flippases and floppases, which also transport phospholipids between adjacent bilayers, scramblases do so in a manner that is both ATP-independent and bidirectional. Therefore, they can be viewed as transporters involved in the facilitated diffusion of phospholipids, while flippases and floppases are more similar to proteins that perform active transport (Fig. 2) (Chen, Ding *et al.* 2022). The importance of scramblase proteins lies in membrane dynamics, which are necessary for organelle formation and transport, making them essential for normal cell function.

TMEM41B and VMP1 are ER-resident membrane proteins that share an evolutionarily conserved domain named the VTT (VMP1, TMEM41B and Tvp38) domain homologous to regions in a host of other bacterial DedA family proteins and thus part of the DedA superfamily (48, 49). The protein structure of the DedA/VTT domain was predicted using the protein structure prediction software trRosetta as consisting of four transmembrane helices along with two reentrant loops, which are helical domains that only extend partway through the lipid membrane before turning and exiting from the same side (50, 51). Later, a Substituted Cysteine Accessibility Method (SCAM) was used to validate the structure, which helped identify regions of the protein facing the ER cytosol versus the lumen. These results confirmed the four transmembrane helices and two reentrant loops of TMEM41B. Analysis of other protein structures showed that structures with dual reentrant loops are common in transporters like aquaporins and ion channels, where the reentrant loops are directly involved in substrate binding (51). In protein structures generated by AlphaFold, the dual-reentrant loops and the four transmembrane helices of the DedA domain can be seen in high-confidence prediction regions of HsTMEM41B, although low-confidence predictions within the DedA domain of the HsVMP1 model make interpretation more challenging (49).

Structural modeling of homologous proteins in prokaryotes, along with consideration of TMEM41B’s role in the ER-Golgi shuttle, led to the hypothesis that TMEM41B functions as a lipid scramblase. It is thought to facilitate bidirectional, ATP-independent phospholipid movement, balancing lipids between the inner and outer leaflets of lipid bilayers. The phospholipid scramblase activity of TMEM41B and VMP1 was demonstrated experimentally using a dithionite assay. In this assay, fluorescence-tagged liposomes were treated with dithionite, a reducing agent that cannot cross the liposome membrane. Without scramblases, dithionite treatment results in an expected 50% decrease in fluorescence under control conditions. When recombinant TMEM41B or VMP1 were added to the liposomes, a 90% decrease in fluorescence was observed, confirming their lipid transport activity and establishing TMEM41B and VMP1 as scramblases (21, 23, 52).

## Scramblases regulating the dynamics of the autophagosomal membrane and autophagy

Macroautophagy (hereafter called autophagy) is a highly conserved lysosome-mediated degradation process that supports cell adaptive survival during stresses like starvation (5, 53). A key feature of autophagy is the dynamic remodeling of membranes to form double-membraned autophagosomes. Although the precise sources of membranes for autophagosome formation are not fully known, it is widely accepted that autophagosomes originate from isolation membranes (IMs), also called phagophores, near-specific areas of the ER that contain double-FYVE-containing protein 1 (DFCP1). The IM is then nucleated, elongated, and closed to create double-membraned autophagosomes, which eventually fuse with lysosomes to form autolysosomes, leading to the degradation of autophagosome contents and inner membranes by lysosomal hydrolases (5, 54, 55, 56). While multiple autophagy-related (ATG) proteins have been identified and extensively studied in regulating the autophagy process (56, 57), little is known about the role of lipid compositions of the autophagosome membrane in shaping and remodeling these structures during autophagy.

VMP1 was first identified as an acute pancreatitis-associated protein that may interact with BECLIN-1 to regulate the PI3 kinase complex for initiating autophagosome formation (58). Additional studies show that VMP1 interacts with sarcoendoplasmic reticulum calcium-ATPase (SERCA) calcium pump and inhibits the contact between the IM and the ER by promoting SERCA activity. Loss of VMP1 increases the association of IMs with the ER, resulting in the blockage of autophagosome formation.

The role of TMEM41B in autophagy regulation was identified through three separate genome-wide genetic screens (59, 60, 61). These studies demonstrate that TMEM41B is necessary for autophagosome closure, as autophagosome formation is halted, leading to the accumulation of small LC3-II-positive IMs in TMEM41B-KO cells. Under normal autophagic flux, LC3-II-decorated autophagosomes fuse with lysosomes to form autolysosomes, where lysosomal proteases degrade the inner membrane LC3-II, and ATG4B deconjugates the outer membrane LC3-II, reducing its levels (5, 53, 56). Both LC3-II and the autophagy substrate protein p62 levels increase in *VMP1* KO and *TMEM41B* KO cells, indicating that VMP1 and TMEM41B have similar roles in autophagosome closure regulation. TMEM41B directly interacts with VMP1 both in vivo and in vitro, and functions upstream of VMP1 in autophagy regulation, as overexpression of VMP1 rescues autophagy defects in *TMEM41B* KO cells, while overexpression of TMEM41B cannot rescue defects in *VMP1* KO cells (59).

In addition, TMEM41B and VMP1 work alongside another autophagosome scramblase, ATG9A, in regulating autophagy. ATG9A is the only known genuine ATG protein with transmembrane domains that have scramblase activity (62). ATG9A is specific to the ATG9A vesicles that originate from the Golgi apparatus in a process requiring ATG23 and ATG27 in yeast, which was thought to be the only membrane source during early autophagosome formation (63, 64). ATG9A contributes to the expansion of the ER-associated autophagosome membrane alongside ATG2, a member of the lipid transfer proteins (LTPs). In addition to regulating autophagosome membrane expansion, ATG9A partners with scaffold protein IQGAP1 (mainly functioning for cytoskeletal dynamics) and the key ESCRT-III component CHMP2A to facilitate the final stage of autophagosome closure (65). While both VMP1 and TMEM41B may regulate autophagy at the same step by promoting the elongation and closure of autophagosomes, they may also have distinctive non-redundant roles, as single deletion of either VMP1 or TMEM41B in cells is sufficient to block autophagic flux, despite the presence of the other proteins in both cultured cells and in mouse livers (59, 66, 67). These observations also suggest that TMEM41B and VMP1 may have distinct roles in regulating autophagy from ATG9A as ATG9A cannot compensate for the loss of either TMEM41B or VMP1.

LTPs are characterized by their ability to enable lipid transfer between membranes and function at membrane contact sites (MCSs), bridging adjacent membranes to allow the direct transfer of lipid molecules without requiring vesicular transport (68). Such localization in MCS makes lipid transport not only quick and efficient but also specific. LTPs are essential for maintaining cellular lipid homeostasis and are irreplaceable for various cellular processes, including signaling, membrane dynamics, and lipid metabolism (69). LTPs consist of two main structural parts: membrane tethering domains and lipid transport modules. The membrane tethering domains attach the LTPs to specific membrane sites, bringing them into close proximity. Next to these domains is the lipid transport module, which is crucial according to the "shuttle model" of lipid transfer (70, 71). This modular organization and mechanism of action emphasize the specialized role of LTPs in cellular lipid regulation and highlight their importance in maintaining lipid homeostasis across different cellular compartments (72).

ATG2 has a lipid-transfer-protein-like hydrophobic cavity that holds phospholipid acyl chains to facilitate phospholipid transfer and provide them for autophagosome formation (73). ATG2 transports lipid molecules from the outer leaflet of a donor membrane to the outer leaflet of an acceptor membrane, such as autophagosomal membranes, causing an imbalance in the number of lipid molecules between the outer and inner leaflets of autophagosomes. To fix this imbalance, the lipid scramblase ATG9A moves lipids from the outer to the inner leaflet of autophagosomes (74). ATG2 also acts as a component in the ATG18-ATG2 complex, which tethers the isolation membrane to the ER and transports lipids from the cytoplasmic leaflet of the ER to that of the isolation membrane (75). The scramblase ATG9A then shuttles these new lipids into the luminal leaflet. VMP1 and TMEM41B directly bind to ATG2, while TMEM41B also interacts with ATG9A depending on ATG2 (52). This process transfers lipids between the cytosolic leaflets of neighboring organelles, with scramblases balancing the leaflets of donor and acceptor membranes as lipids are either removed or supplied. Therefore, TMEM41B and VMP1 can collaborate with LTPs, which are crucial for maintaining lipid balance and membrane behavior during cellular processes like autophagosome formation (Fig. 2).

**Fig. 3:**
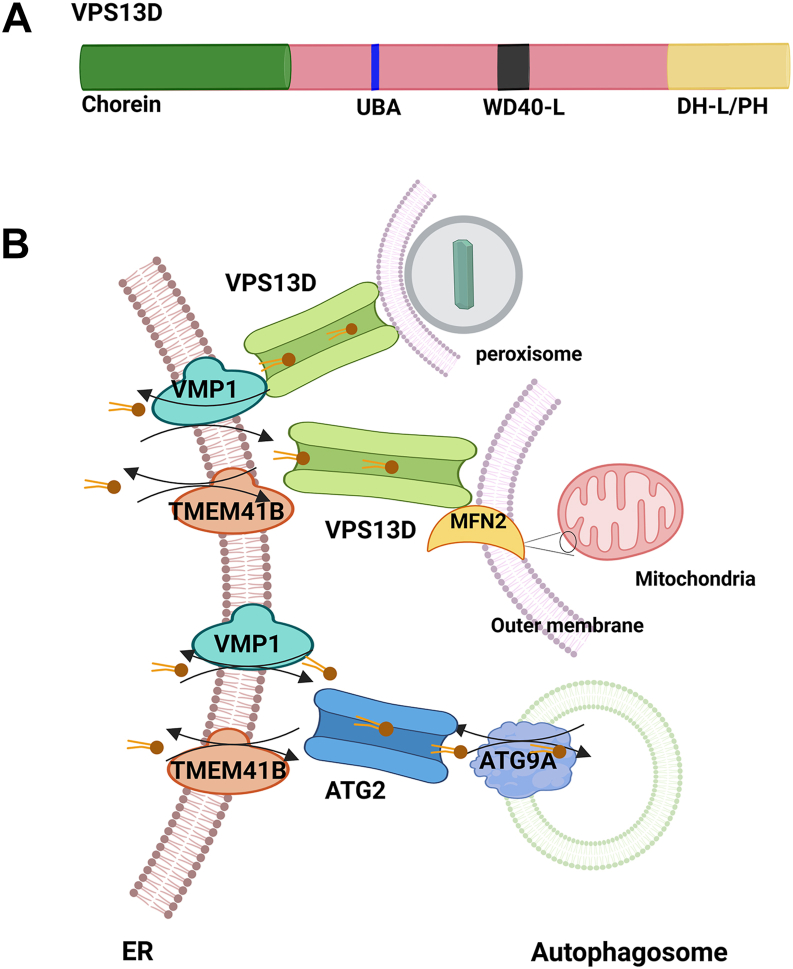
A diagram showing the coordination of scramblases and LTPs in the phospholipid transport, membrane contact and organelle biogenesis. A: VPS13D domain organization. VPS13D has a Chorein-N domain at the N-terminal, a Ubiquitin-associated (UBA) domain, a Dbl homology-like (DH-L)/pleckstrin homology domain (PH). B: VPS13D regulates ER-mitochondrial and peroxisome contact, as well as mitochondrial and peroxisome functions. VPS13D acts downstream of VMP1 to transfer phospholipids between ER bilayers and the outer mitochondrial membrane, controlling mitochondrial-ER contact, mitochondrial morphology, and mitophagy that require the mitochondrial fusion protein mitofusin 2 (MFN2). VPS13D also transports lipids to peroxisomes to promote their proliferation. Additionally, the N-terminal region of ATG2 is anchored to the ER through interactions with ER-resident scramblases VMP1 and/or TMEM41B, which move phospholipids between bilayers in an ATP-independent manner. ATG2 then transfers phospholipids (yellow) from the ER to the expanding autophagosomal membranes, where ATG9A functions as a lipid scramblase to transfer lipids between membrane leaflets.

In addition to ATG2, vacuolar protein sorting-associated protein 13 (VPS13) family proteins are also LTPs that may work with TMEM41B and VMP1 to regulate lipid homeostasis. The VPS13 family includes four members in humans: VPS13A, B, C, and D. The *VPS13* genes were first identified through yeast genetic screens designed to find genes involved in membrane trafficking, especially at the interface between the Golgi complex and the vacuole, with the goal of understanding the molecular mechanisms that control intracellular transport pathways and organelle communication (76). A loss-of-function mutation in VPS13A is associated with chorea-acanthocytosis, a complex neurodegenerative disorder (77). Similarly, mutations in VPS13B have been identified as the cause of Cohen syndrome, a rare neurodevelopmental disorder (78). Furthermore, the role of VPS13C has been implicated in Parkinson’s disease (79), and mutations in VPS13D are associated with a range of neurological conditions (80). The VPS13 family shares a similar structure that includes a chorein-N domain, an FFAT motif, a WD40-like domain, and a DH-L/PH domain containing an ATG-C homology motif (81). The Chorein-N domain of VPS13 has a scoop-like shape with a hydrophobic, concave surface designed for binding glycerolipid tails. This structural feature, combined with VPS13’s localization at MCS, indicates the protein functions as a bridge for lipid transfer between neighboring bilayers, aiding their movement along its groove (82). VPS13A, VPS13C, and VPS13D specifically localize at contact sites between the ER and other organelles such as mitochondria and peroxisomes, which serve as lipid conduits to transfer lipids to mitochondria and peroxisomes (83, 84). In *Drosophila* intestinal cells, VMP1 functions upstream of VPS13D to regulate MCS, mitochondrial shape, and mitophagy. Disruptions in this pathway, especially involving VPS13D, can lead to problems with autophagy, mitochondrial structure, and human movement disorders (85). Therefore, the current model suggests that LTPs and scramblases work together to facilitate membrane expansion and organelle biogenesis. LTPs, such as those in the VPS13 family, transfer lipids between organelles, while scramblases like TMEM41B, VMP1, and ATG9A help maintain lipid distribution across the two membrane leaflets. This partnership allows for efficient and regulated lipid transfer, supporting the growth and formation of new organelles, such as autophagosomes, mitochondria, and peroxisomes, to help maintain cellular homeostasis (86, 87) (Fig. 3). However, the role of LTPs in VLDL secretion has not been studied.

## Scramblase and membrane repair

In addition to regulating autophagy, recent evidence shows that ATG9A plays a role in protecting the plasma membrane and lysosomal membrane from damage independent of its role in autophagy (88, 89, 90). ATG9A is found on vesicles that traffic to the plasma membrane and interacts with IQGAP1, which coordinates calcium signaling and ESCRT proteins to mediate plasma membrane repair (88). For lysosomal membrane repair, ATG9A vesicles containing ADP ribosylation factor interacting protein 2 (ARFIP2) bind and sequester PI4P on lysosomes, balancing lipid transfer by oxysterol-binding protein-like proteins (OSBPL) and promoting lysosomal membrane repair and homeostasis after damage and bacterial infection. The ATG9A scramblase activity is not required for plasma membrane repair in human cells or lysosomal repair in worms, but it is necessary for lysosomal membrane repair in human cells (88, 89, 90). This suggests that ATG9 may have different functions in lysosome repair between worms and humans, as well as between plasma membrane and lysosomal membrane repair in humans. Notably, TMEM16F, a calcium-activated lipid scramblase, induced rapid “lipid scrambling” in the plasma membrane to repair the plasma membrane that was damaged by pore-forming agents (91), further supporting the notion that lipid scramblase may help repair membrane damage. Interestingly, instead of repairing the membrane, the family of X Kell-related (XKR) plasma membrane scramblases functions to promote the externalization of phosphatidylserine on the surface of apoptotic cells for their recognition and clearance by macrophages (92). Since TMEM41B and VMP1 are mainly located at the ER membrane and mitochondria-ER contact sites, they are less likely to be involved in plasma membrane and lysosomal membrane repair or in the externalization of phosphatidylserine. This may also explain why only VMP1 and TMEM41B are known to regulate APOB lipoproteins, as the ER is the primary site for the biogenesis of these lipoproteins. Therefore, it is reasonable to suggest that the subcellular locations of various scramblases could account for their specific functions, which include lipoprotein biogenesis, autophagosome formation, and the repair of organelles and the plasma membrane. Overall, these recent exciting findings highlight a complex role of scramblases in cell biology by regulating membrane dynamics and repair through changing and balancing phospholipid levels between membrane leaflets.

## Other functions of VMP1 and TMEM41B

In addition to regulating autophagy, both VMP1 and TMEM41B are necessary for β-coronavirus infection, functioning at different stages of double-membrane vesicle biogenesis that are essential for viral genome replication and transcription (93). TMEM41B has also been found to be crucial in mediating flavivirus and coronavirus infection, although its role in autophagy may not be relevant to its function in viral infection (94, 95). VMP1 also inhibits ER contacts with other organelles such as mitochondria, lipid droplets, and endosomes/endolysosomes in cultured COS7 cells, likely by regulating local Ca^2+^ levels around the MCSs to cause their disassembly (96). Finally, emerging evidence indicates that VMP1 and TMEM41B also play vital roles in lipoprotein formation and VLDL secretion, as discussed in detail below.

## TMEM41B and VMP1 in regulating VLDL Secretion

In addition to autophagy, VMP1 and TMEM41B have recently been found to have important roles in VLDL secretion. Loss of hepatic VMP1 and TMEM41B has been found to impair hepatic lipoprotein secretion, resulting in rapid development of steatohepatitis in mice (23, 67, 97). Electron microscopy analysis provides strong evidence that the loss of VMP1 causes neutral lipids to become trapped within the lipid bilayer of the ER membrane, halting lipoprotein budding into the ER lumen (22, 66). These LDs displayed clear electron-dense edges, likely representing the surrounding phospholipid monolayer around the lipid structures, and may be called “ER-phospholipid bilayer LDs,” found in both *Vmp1* KO and *Tmem41b* KO hepatocytes (66, 67). Confocal microscopy also shows that most LDs in *Vmp1* KO hepatocytes are positive for APOB and KDEL (an ER marker) but negative for perilipin 2 (a cytosolic LD marker). Interestingly, recent EM data from us, now published in a preprint, revealed that a small portion of the LDs was located within the ER lumen (67). These small, luminal LD-like structures are likely composed of pre-mature VLDL and may correspond to the same APOB-positive LDs observed by confocal microscopy. Moreover, liver lipid fractions from *Vmp1* KO mouse livers showed enrichment with calnexin (an ER marker) but less perilipin-2 and almost no detectable SEC24D (a COPII component) and APOB100 (a lipoprotein), further supporting the idea that *Vmp1* deficiency causes the accumulation of ER bilayer LDs, not cytosolic LDs (66). However, both EM and confocal microscopy in these studies have clear limitations. The EM data were not quantitative, and even the quantitative confocal data relied on a small number of cells. Future studies using immune-gold EM with APOB antibody would give more definitive evidence for these “LD” structures in *Vmp1* KO and *Tmem41b* KO cells. Better techniques to distinguish LDs and nascent VLDL at the subcellular level will also help further characterize these LD structures and clarify the key steps of VLDL formation seen in *Vmp1* KO and *Tmem41b* KO cells.

Loss of hepatic VMP1 did not change the hepatic levels of MTP or PDI, implicating that MTP and PDI are less likely to participate in retaining neutral lipids in the ER membrane bilayer of *Vmp1* KO hepatocytes. Phospholipids, especially the levels of phosphatidylcholine (PC) and phosphatidylethanolamine (PE), as well as the fatty acyl chain compositions of these lipids, are essential for the assembly and secretion of VLDL. Loss of VMP1 decreased hepatic PC and PE content and altered the acyl chain composition of phospholipids. ER-mitochondria contact sites are enriched with phospholipid synthesis enzymes, including phosphatidylserine decarboxylase (PISD) and phosphatidylethanolamine N-methyltransferase (PEMT). While loss of VMP1 has been reported to increase ER-mitochondria contact sites in cultured COS7 cells, ER-mitochondrial contact sites markedly decreased *Vmp1* KO hepatocytes (66, 96). Therefore, it appears that organelle contact regulation by VMP1 may vary depending on the cell type. However, how VMP1 controls ER-mitochondria and other organelle contact sites, as well as their roles in phospholipid synthesis, remains to be further investigated. Nonetheless, it is likely that VMP1 may influence phospholipid biosynthesis by regulating ER-mitochondrial contact independently of its scramblase activity. Reduced PC and PE levels, along with altered acyl chain composition of phospholipids, may thus modify the biophysical tension and curvature of the ER membrane, which can prevent the import of neutral lipids from the ER membrane bilayer into the ER lumen. Loss of VMP1 resulted in reduced expression of PISD and PEMT, two essential enzymes involved in PE and PC synthesis (67). This may partly explain the lower hepatic levels of PC and PE observed in *Vmp1* KO mice. Reduced PC and PE contents, along with altered acyl chain composition of phospholipids, may thus affect the biophysical tension and curvature of the ER membrane, which halts the import of neutral lipids from the ER membrane bilayer into the ER lumen (66). Further research is needed to understand how VMP1 influences PISD and PEMT levels in the liver.

As we discussed earlier, the COPII complex facilitates vesicle formation at ER exit sites, enabling the transport of VLDL from the ER to the Golgi for further processing and eventual secretion. Immunoprecipitation and confocal microscopy analysis revealed that VMP1 directly interacts with and colocalizes with SEC24D. Loss of VMP1 resulted in reduced levels of SEC23A, SEC24C, and SEC24D (66), which are key components of COPII in mouse livers. More significant decreases of SAR1A, SEC24A, SEC24D, and SEC23A were observed in *Tmem41b* KO mouse livers compared to *Vmp1* KO mouse livers, indicating that TMEM41B appears to be a more critical regulator of COPII-coated vesicle transport at the ER exit sites than VMP1 (67). Interestingly, the levels of SAR1A and SAR1B remained unaffected in hepatocyte-specific *Vmp1* KO mouse livers (66, 67). TMEM41B directly interacts with SURF4 and APOB, while VMP1 interacts with APOB to a lesser extent than TMEM41B (23). These findings indicate that VMP1 and TMEM41B may directly engage with the COPII complex and ER exit components to stabilize COPII proteins and regulate VLDL exit from the ER and trafficking to the Golgi.

As discussed earlier, both VMP1 and TMEM41B share similar protein structures and are located in the ER, with phospholipid scramblase activity (21, 23). Deletion of *Tmem41b* in mouse livers using sgRNAs, or AAV8-TBG-Cre and Alb-Cre mediated deletion of *Tmem41b* in *Tmem41b* flox/flox mice, all lead to severe defects in VLDL secretion and rapid development of MASH (23, 67). Double deletion of VMP1 and TMEM41B in mouse livers showed similar defects in VLDL secretion and MASH development (67), suggesting that TMEM41B and VMP1 have similar roles in regulating hepatic VLDL secretion. Despite these similarities, some differences were noted between TMEM41B and VMP1. TMEM41B exhibited strong direct interactions with SURF4 and APOB, whereas VMP1 showed weaker binding with APOB, and no direct interaction with SURF4 was detected (23). These findings lead us to propose a model where VMP1 acts upstream to prevent lipoproteins from entering the ER lumen, while TMEM41B may act downstream to block their exit, similar to the function of SURF4. However, no lipoprotein particles were observed in the ER lumen of hepatocytes with *Tmem41b* depletion by sgRNA in mouse livers, which was quite unexpected. The authors suggest that the loss of TMEM41B significantly increases ER membrane curvature, potentially leading to the absence of ER luminal lipoprotein (LD) (23). Interestingly, LDs trapped within ER bilayer were observed in *Tmem41b* KO mouse livers using AAV8-TBG-Cre or Alb-Cre approaches to delete *Tmem41b* (67). It is likely that the deletion efficiency of AAV8-TBG-Cre or Alb-Cre is higher than that achieved with sgRNA. Further analysis showed decreased APOB100 proteins in *Tmem41b* KO mouse livers, supporting the absence of lipidated APOB100 (23). This, however, does not definitively distinguish whether these are ER bilayer or luminal LDs, as both could result in the lack of mature lipoproteins. Indeed, decreased APOB100 proteins are also observed in *Vmp1* KO mouse livers (66). Notably, when the VLDL secretion was directly compared in liver-specific *Vmp1* KO, *Tmem41b* KO and *Vmp1*, *Tmem41b* double KO (DKO) mice, *Vmp1* KO mice had similar VLDL secretion with the DKO mice but showed more severe VLDL secretion defects compared with *Tmem41b* KO mice. Results from the hepatic lipidomics analysis showed much lower hepatic PC and PE levels in *Vmp1* KO mice than in *Tmem41b* KO mice (67), consistent with the observation that *Vmp1* KO mice had more severe VLDL secretion defects.

Because deletion of either *Tmem41b* or *Vmp1* alone is enough to cause the VLDL secretion defects and MASH, which may suggest that the presence of one cannot compensate for the loss of the other. However, the levels of VMP1 did not change in *Tmem41b* KO mice, whereas the levels of TMEM41B were lower in *Vmp1* KO mice (67), suggesting that VMP1 and TMEM41B may stabilize with each other, likely due to their direct interactions (98). Overexpressing *Vmp1* or *Tmem41b* in mouse livers deficient in either gene partially reduces steatosis and corrects autophagy defects, indicating a functional interaction between TMEM41B and VMP1 in regulating hepatic VLDL secretion and autophagy. However, TMEM41B's ability to restore VLDL secretion and autophagy in *Vmp1*-deficient livers depends on gene dosage. In *Tmem41b*-deficient livers, both low and high levels of VMP1 improve VLDL secretion and steatosis, but the high levels VMP1 fail to correct autophagy defects, highlighting the distinct roles of VMP1 and TMEM41B (67). Although both TMEM41B and VMP1 proteins have a DedA domain with two transmembrane helices, one extramembrane helix, and two reentrant loops, the main structural difference is that TMEM41B has fewer transmembrane domains, while VMP1 lacks an extramembrane domain (48, 49). Whether these structural differences contribute to variations in their regulation of hepatic VLDL secretion, liver injury, and autophagy remains to be explored in future studies.

In addition to affecting the liver, deleting *Vmp1* in the small intestine using Villin Cre led to the accumulation of neutral lipids in intestinal epithelial cells in mice. Interestingly, serum levels of cholesterol and lipoproteins such as high-density lipoprotein (HDL) decreased, but serum triglyceride and low-density lipoprotein (LDL) levels remained unchanged in the intestine-specific *Vmp1* KO mice compared to matched wild-type mice. Deleting *VMP1* in human hepatoma HepG2 cells resulted in reduced secretion of APOB, APOE (a component of VLDL), and APOA-I (a component of HDL) (22). Additionally, decreased serum total cholesterol levels were observed in liver-specific *Vmp1* KO mice (66). Similar decreases in serum cholesterol and HDL levels were seen in liver-specific *Tmem41b* KO mice (23). These findings suggest that VMP1 or TMEM41B may also regulate HDL cholesterol, in addition to APOB-mediated VLDL secretion. Future studies are needed to determine whether VMP1 and TMEM41B influence APOB-VLDL and APOA-I-HDL through similar or different mechanisms in the liver.

## Autophagy and VLDL Secretion in MASH

While TMEM41B and VMP1 regulate both autophagy and VLDL secretion, it is most likely that these two cellular processes occur independently. Large lipid-containing structures were not observed in zebrafish lacking the *rb1cc1/fip20* or *atg5* gene (22). Moreover, deletion of hepatic *Atg5* or *Atg*7, the essential autophagy-related gene, does not cause steatosis in mice, indicating that blocking autophagy is dispensable for steatosis development (99, 100). Loss of hepatic *Vmp1* increased hepatic accumulation of acylcarnitines, along with decreased ketone bodies and ^14^C-palmitate oxidation, indicating reduced fatty acid oxidation (FAO) (66). Similar reductions in FAO were observed in *Vps15*, *Atg7*, and *Atg5* KO mice. However, as discussed earlier, liver-specific *Atg5* and *Atg7* KO mice show normal VLDL secretion and do not display obvious hepatic steatosis, suggesting that decreased FAO may not be the main cause of lipid buildup in *Vmp1* KO mouse livers. Therefore, hepatic steatosis in *Vmp1* KO mice is likely primarily due to impaired VLDL secretion rather than decreased FAO and is probably independent of autophagy deficits. Increased cell death, inflammation, and fibrosis have been observed in hepatic *Atg5* and *Atg7*-deficiency mice, but not impaired VLDL secretion and steatosis (99, 101, 102, 103). Intriguingly, most genetically modified mouse MASLD models, such as hepatic *Surf4-, Lpcat3-*, *Mea6-*, and *Sar1b*-deficiency mice, showed increased hepatic steatosis with defective hepatic VLDL secretion without altered AOPB100 but none of them developed MASH (28, 104, 105, 106). Therefore, the MASH phenotypes in *Vmp1* and *Tmem41b* KO mice differ from those in MASLD models. The MASH phenotypes in *Vmp1* and *Tmem41b* KO mice probably result from combined impairments in VLDL secretion and autophagy, which are specific to TMEM41B and VMP1.

## Conclusions and future perspectives

While many new pharmacologic therapies are being developed to specifically target MASLD/MASH through regulation of hepatocyte-specific cellular receptors, not all aspects of hepatic lipid regulation have been fully explored. Liver lipid homeostasis is controlled broadly through several key processes: lipid uptake, lipid secretion, lipid oxidation, and de novo lipogenesis, with most drug targets, such as FGF21 and THRβ, regulating multiple of the processes simultaneously. While most cases of hepatic steatosis likely result from metabolic dysfunction involving chronic caloric surplus and reduced physical activity, a small number of individuals, particularly in such cases as “lean MASH,” may have secretion-reducing polymorphisms such as *Tm6sf2*, which have been found to increase susceptibility to MASLD/MASH while being somewhat protective against atherosclerosis. In these cases, restoration of secretion may be indicated. Therein lies the potential strength of scramblase-targeted therapy in regulating individual processes, allowing for tight control of lipid homeostasis and individualized therapy.

The mechanisms involved in lipoprotein secretion and transport throughout circulation involve a complex set of processes with multiple enzymes and proteins responsible for lipid-protein conjugation, trafficking, vesicle formation within cells, and hydrolysis and reuptake within the serum to mobilize these energy stores. Although the roles of some key players, including APOB, MTP, PDI, SAR1, and COP proteins, have been well described, many others, including TMEM41B and VMP1, are emerging as new key players for further investigation. The relationship between scramblase proteins and hepatic steatosis or lipid secretion has been only sparsely studied, despite the importance of membrane remodeling in lipid mobilization and trafficking. This may partly be due to the more “passive” nature of scramblase proteins, which resemble facilitated transport vesicles more than active cellular receptors. Hepatic VMP1 and TMEM41B levels decreased in MASH livers, and overexpression of VMP1 in a diet-induced MASH model in mice restored the VLDL secretion defect (97), underscoring the potential of targeting these scramblases for MASH treatment.

Intronic single-nucleotide polymorphisms (SNPs) in the *VMP1* gene have been identified and are linked to increased levels of circulating LDL cholesterol, total cholesterol, and triglycerides, or decreased levels of lipoprotein-associated phospholipase A2, according to human genome-wide association studies (107, 108). SNPs in the *TMEM41B* gene are connected to a reduced ability of cells to support infections by flaviviruses (such as Zika and yellow fever) and coronaviruses (such as SARS-CoV-2) (109, 110), although no association with circulating lipid changes has been reported yet for *TMEM41B* SNPs. While more human-related evidence is needed to establish a link between lipid metabolism and VMP1 and TMEM41B, the lower circulating triglycerides and cholesterol observed in *Vmp1* and *Tmem41b* knockout mice may also offer new insights into potential strategies for preventing cardiovascular disease and atherosclerosis by targeting the scramblases VMP1 and TMEM41B. Future research on TMEM41B and VMP1 may investigate whether manipulating membrane phospholipid balance through these proteins influences lipid secretion rates and lipoprotein lipid content, and potentially enhances autophagy. If so, modulating VLDL secretion and autophagy could be an effective target for scramblase-based MASLD/MASH therapy.

## Conflict of interest

The authors declare that they do not have any conflicts of interest with the content of this article.

## References

[1] Eslam M., George J. (2023). Two years on, a perspective on MAFLD. eGastroenterology.

[2] Rinella M.E., Sookoian S. (2024). From NAFLD to MASLD: updated naming and diagnosis criteria for fatty liver disease. J. Lipid Res..

[3] Wang X., Zhang L., Dong B. (2025). Molecular mechanisms in MASLD/MASH-related HCC. Hepatology.

[4] Younossi Z.M., Kalligeros M., Henry L. (2025). Epidemiology of metabolic dysfunction-associated steatotic liver disease. Clin. Mol. Hepatol..

[5] Mizushima N., Levine B., Cuervo A.M., Klionsky D.J. (2008). Autophagy fights disease through cellular self-digestion. Nature.

[6] Karim G., Bansal M.B. (2023). Resmetirom: an orally administered, smallmolecule, liver-directed, β-selective THR agonist for the treatment of non-alcoholic fatty liver disease and non-alcoholic steatohepatitis. Touchrev. Endocrinol..

[7] Jara M., Norlin J., Kjaer M.S., Almholt K., Bendtsen K.M., Bugianesi E. (2025). Modulation of metabolic, inflammatory and fibrotic pathways by semaglutide in metabolic dysfunction-associated steatohepatitis. Nat. Med..

[8] Sanyal A.J., Newsome P.N., Kliers I., Ostergaard L.H., Long M.T., Kjær M.S. (2025). Phase 3 trial of semaglutide in metabolic dysfunction-associated steatohepatitis. N. Engl. J. Med..

[9] Steinberg G.R., Valvano C.M., De Nardo W., Watt M.J. (2025). Integrative metabolism in MASLD and MASH: pathophysiology and emerging mechanisms. J. Hepatol..

[10] Carli F., Della Pepa G., Sabatini S., Vidal Puig A., Gastaldelli A. (2024). Lipid metabolism in MASLD and MASH: from mechanism to the clinic. JHEP Rep..

[11] Zhang L., Wang X., Chen X.W. (2025). The biogenesis and transport of triglyceride-rich lipoproteins. Trends Endocrinol. Metab..

[12] van Zwol W., van de Sluis B., Ginsberg H.N., Kuivenhoven J.A. (2024). VLDL biogenesis and secretion: it takes a village. Circ. Res..

[13] Feingold K.R. (2022). Lipid and lipoprotein metabolism. Endocrinol. Metab. Clin. North Am..

[14] Kurano M., Tsukamoto K., Hara M., Ohkawa R., Ikeda H., Yatomi Y. (2015). LDL receptor and ApoE are involved in the clearance of ApoM-associated sphingosine 1-phosphate. J. Biol. Chem..

[15] Borén J., Graham L., Wettesten M., Scott J., White A., Olofsson S.O. (1992). The assembly and secretion of ApoB 100-containing lipoproteins in Hep G2 cells. ApoB 100 is cotranslationally integrated into lipoproteins. J. Biol. Chem..

[16] Mobin M.B., Gerstberger S., Teupser D., Campana B., Charisse K., Heim M.H. (2016). The RNA-binding protein vigilin regulates VLDL secretion through modulation of Apob mRNA translation. Nat. Commun..

[17] Vatandaslar H., Garzia A., Meyer C., Godbersen S., Brandt L.T.L., Griesbach E. (2023). In vivo PAR-CLIP (viP-CLIP) of liver TIAL1 unveils targets regulating cholesterol synthesis and secretion. Nat. Commun..

[18] Zhang Z., Zong C., Jiang M., Hu H., Cheng X., Ni J. (2020). Hepatic HuR modulates lipid homeostasis in response to high-fat diet. Nat. Commun..

[19] Ginsberg H.N., Fisher E.A. (2009). The ever-expanding role of degradation in the regulation of apolipoprotein B metabolism. J. Lipid Res..

[20] Segrest J.P., Jones M.K., Dashti N. (1999). N-terminal domain of apolipoprotein B has structural homology to lipovitellin and microsomal triglyceride transfer protein: a “lipid pocket” model for self-assembly of apoB-containing lipoprotein particles. J. Lipid Res..

[21] Li Y.E., Wang Y., Du X., Zhang T., Mak H.Y., Hancock S.E. (2021). TMEM41B and VMP1 are scramblases and regulate the distribution of cholesterol and phosphatidylserine. J. Cell Biol..

[22] Morishita H., Zhao Y.G., Tamura N., Nishimura T., Kanda Y., Sakamaki Y. (2019). A critical role of VMP1 in lipoprotein secretion. Elife.

[23] Huang D., Xu B., Liu L., Wu L., Zhu Y., Ghanbarpour A. (2021). TMEM41B acts as an ER scramblase required for lipoprotein biogenesis and lipid homeostasis. Cell Metab..

[24] Thierer J.H., Foresti O., Yadav P.K., Wilson M.H., Moll T.O.C., Shen M.C. (2024). Publisher correction: Pla2g12b drives expansion of triglyceride-rich lipoproteins. Nat. Commun..

[25] Li B.T., Sun M., Li Y.F., Wang J.Q., Zhou Z.M., Song B.L. (2020). Disruption of the ERLIN-TM6SF2-APOB complex destabilizes APOB and contributes to non-alcoholic fatty liver disease. PLoS Genet..

[26] Shin J.Y., Hernandez-Ono A., Fedotova T., Ostlund C., Lee M.J., Gibeley S.B. (2019). Nuclear envelope-localized torsinA-LAP1 complex regulates hepatic VLDL secretion and steatosis. J. Clin. Invest..

[27] Ye J., Li J.Z., Liu Y., Li X., Yang T., Ma X. (2009). Cideb, an ER- and lipid droplet-associated protein, mediates VLDL lipidation and maturation by interacting with apolipoprotein B. Cell Metab..

[28] Santos A.J., Nogueira C., Ortega-Bellido M., Malhotra V. (2016). TANGO1 and Mia2/cTAGE5 (TALI) cooperate to export bulky pre-chylomicrons/VLDLs from the endoplasmic reticulum. J. Cell Biol..

[29] Barlowe C., Schekman R. (1993). SEC12 encodes a guanine-nucleotide-exchange factor essential for transport vesicle budding from the ER. Nature.

[30] Weissman J.T., Plutner H., Balch W.E. (2001). The mammalian guanine nucleotide exchange factor mSec12 is essential for activation of the Sar1 GTPase directing endoplasmic reticulum export. Traffic.

[31] Peotter J., Kasberg W., Pustova I., Audhya A. (2019). COPII-mediated trafficking at the ER/ERGIC interface. Traffic.

[32] Loftus A.F., Hsieh V.L., Parthasarathy R. (2012). Modulation of membrane rigidity by the human vesicle trafficking proteins Sar1A and Sar1B. Biochem. Biophys. Res. Commun..

[33] Levy E., Poinsot P., Spahis S. (2019). Chylomicron retention disease: genetics, biochemistry, and clinical spectrum. Curr. Opin. Lipidol..

[34] Bi X., Corpina R.A., Goldberg J. (2002). Structure of the Sec23/24-Sar1 pre-budding complex of the COPII vesicle coat. Nature.

[35] Stagg S.M., Gürkan C., Fowler D.M., LaPointe P., Foss T.R., Potter C.S. (2006). Structure of the Sec13/31 COPII coat cage. Nature.

[36] Bielli A., Haney C.J., Gabreski G., Watkins S.C., Bannykh S.I., Aridor M. (2005). Regulation of Sar1 NH2 terminus by GTP binding and hydrolysis promotes membrane deformation to control COPII vesicle fission. J. Cell Biol..

[37] Hariri H., Bhattacharya N., Johnson K., Noble A.J., Stagg S.M. (2014). Insights into the mechanisms of membrane curvature and vesicle scission by the small GTPase Sar1 in the early secretory pathway. J. Mol. Biol..

[38] Yoshihisa T., Barlowe C., Schekman R. (1993). Requirement for a GTPase-activating protein in vesicle budding from the endoplasmic reticulum. Science.

[39] Bi X., Mancias J.D., Goldberg J. (2007). Insights into COPII coat nucleation from the structure of Sec23.Sar1 complexed with the active fragment of Sec31. Dev. Cell.

[40] Butkinaree C., Guo L., Ramkhelawon B., Wanschel A., Brodsky J.L., Moore K.J. (2014). A regulator of secretory vesicle size, Kelch-like protein 12, facilitates the secretion of apolipoprotein B100 and very-low-density lipoproteins--brief report. Arterioscler Thromb. Vasc. Biol..

[41] Pitman J.L., Bonnet D.J., Curtiss L.K., Gekakis N. (2011). Reduced cholesterol and triglycerides in mice with a mutation in Mia2, a liver protein that localizes to ER exit sites. J. Lipid Res..

[42] Rahim A., Nafi-valencia E., Siddiqi S., Basha R., Runyon C.C., Siddiqi S.A. (2012). Proteomic analysis of the very low density lipoprotein (VLDL) transport vesicles. J. Proteomics.

[43] Wang X., Huang R., Wang Y., Zhou W., Hu Y., Yao Y. (2023). Manganese regulation of COPII condensation controls circulating lipid homeostasis. Nat. Cell Biol..

[44] Takacs C.N., Andreo U., Dao Thi V.L., Wu X., Gleason C.E., Itano M.S. (2017). Differential regulation of lipoprotein and hepatitis C virus secretion by Rab1b. Cell Rep..

[45] Stillemark-Billton P., Beck C., Boren J., Olofsson S.O. (2005). Relation of the size and intracellular sorting of apoB to the formation of VLDL 1 and VLDL 2. J. Lipid Res..

[46] Gusarova V., Brodsky J.L., Fisher E.A. (2003). Apolipoprotein B100 exit from the endoplasmic reticulum (ER) is COPII-dependent, and its lipidation to very low density lipoprotein occurs post-ER. J. Biol. Chem..

[47] Fagone P., Jackowski S. (2009). Membrane phospholipid synthesis and endoplasmic reticulum function. J. Lipid Res..

[48] Hama Y., Morishita H., Mizushima N. (2022). Regulation of ER-derived membrane dynamics by the DedA domain-containing proteins VMP1 and TMEM41B. EMBO Rep..

[49] Chen A., Ding W.X., Ni H.M. (2022). Scramblases as regulators of autophagy and lipid homeostasis: implications for NAFLD. Autophagy Rep..

[50] Mesdaghi S., Murphy D.L., Sánchez Rodríguez F., Burgos-Mármol J.J., Rigden D.J. (2020). In silico prediction of structure and function for a large family of transmembrane proteins that includes human Tmem41b. F1000Res.

[51] Okawa F., Hama Y., Zhang S., Morishita H., Yamamoto H., Levine T.P. (2021). Evolution and insights into the structure and function of the DedA superfamily containing TMEM41B and VMP1. J. Cell Sci..

[52] Ghanbarpour A., Valverde D.P., Melia T.J., Reinisch K.M. (2021). A model for a partnership of lipid transfer proteins and scramblases in membrane expansion and organelle biogenesis. Proc. Natl. Acad. Sci. U.S.A..

[53] Qian H., Chao X., Williams J., Fulte S., Li T., Yang L. (2021). Autophagy in liver diseases: a review. Mol. Aspects Med..

[54] Carlsson S.R., Simonsen A. (2015). Membrane dynamics in autophagosome biogenesis. J. Cell Sci..

[55] Shibutani S.T., Yoshimori T. (2014). A current perspective of autophagosome biogenesis. Cell Res..

[56] Ding W.X., Ma X., Kim S., Wang S., Ni H.M. (2024). Recent insights about autophagy in pancreatitis. eGastroenterology.

[57] He C., Klionsky D.J. (2009). Regulation mechanisms and signaling pathways of autophagy. Annu. Rev. Genet..

[58] Molejon M.I., Ropolo A., Lo Re A., Boggio V., Vaccaro M.I. (2013). The VMP1-Beclin 1 interaction regulates autophagy induction. Sci. Rep..

[59] Morita K., Hama Y., Izume T., Tamura N., Ueno T., Yamashita Y. (2018). Genome-wide CRISPR screen identifies TMEM41B as a gene required for autophagosome formation. J. Cell Biol..

[60] Moretti F., Bergman P., Dodgson S., Marcellin D., Claerr I., Goodwin J.M. (2018). TMEM41B is a novel regulator of autophagy and lipid mobilization. EMBO Rep..

[61] Shoemaker C.J., Huang T.Q., Weir N.R., Polyakov N.J., Schultz S.W., Denic V. (2019). CRISPR screening using an expanded toolkit of autophagy reporters identifies TMEM41B as a novel autophagy factor. PLoS Biol..

[62] Matoba K., Kotani T., Tsutsumi A., Tsuji T., Mori T., Noshiro D. (2020). Atg9 is a lipid scramblase that mediates autophagosomal membrane expansion. Nat. Struct. Mol. Biol..

[63] Noda N.N. (2021). Atg2 and Atg9: intermembrane and interleaflet lipid transporters driving autophagy. Biochim. Biophys. Acta Mol. Cell Biol. Lipids.

[64] Yamamoto H., Kakuta S., Watanabe T.M., Kitamura A., Sekito T., Kondo-Kakuta C. (2012). Atg9 vesicles are an important membrane source during early steps of autophagosome formation. J. Cell Biol..

[65] Javed R., Mari M., Trosdal E., Duque T., Paddar M.A., Allers L. (2025). ATG9A facilitates the closure of mammalian autophagosomes. J. Cell Biol..

[66] Jiang X., Fulte S., Deng F., Chen S., Xie Y., Chao X. (2022). Lack of VMP1 impairs hepatic lipoprotein secretion and promotes nonalcoholic steatohepatitis. J. Hepatol..

[67] Chen A., Nguyen K., Jiang X., Yu X., Xie Y., Liu W. (2025). Overlapping yet distinct functions of VMP1 and TMEM41B in modulating hepatic lipoprotein secretion and autophagy. bioRxiv.

[68] Wong L.H., Gatta A.T., Levine T.P. (2019). Lipid transfer proteins: the lipid commute via shuttles, bridges and tubes. Nat. Rev. Mol. Cell Biol..

[69] Chiapparino A., Maeda K., Turei D., Saez-Rodriguez J., Gavin A.C. (2016). The orchestra of lipid-transfer proteins at the crossroads between metabolism and signaling. Prog. Lipid Res..

[70] Bian X., Zhang Z., Xiong Q., De Camilli P., Lin C. (2019). A programmable DNA-origami platform for studying lipid transfer between bilayers. Nat. Chem. Biol..

[71] Toulmay A., Prinz W.A. (2011). Lipid transfer and signaling at organelle contact sites: the tip of the iceberg. Curr. Opin. Cell Biol..

[72] Reinisch K.M., Prinz W.A. (2021). Mechanisms of nonvesicular lipid transport. J. Cell Biol..

[73] Osawa T., Kotani T., Kawaoka T., Hirata E., Suzuki K., Nakatogawa H. (2019). Atg2 mediates direct lipid transfer between membranes for autophagosome formation. Nat. Struct. Mol. Biol..

[74] Maeda S., Yamamoto H., Kinch L.N., Garza C.M., Takahashi S., Otomo C. (2020). Structure, lipid scrambling activity and role in autophagosome formation of ATG9A. Nat. Struct. Mol. Biol..

[75] Obara K., Sekito T., Niimi K., Ohsumi Y. (2008). The Atg18-Atg2 complex is recruited to autophagic membranes via phosphatidylinositol 3-phosphate and exerts an essential function. J. Biol. Chem..

[76] Rothman J.H., Stevens T.H. (1986). Protein sorting in yeast: mutants defective in vacuole biogenesis mislocalize vacuolar proteins into the late secretory pathway. Cell.

[77] Rampoldi L., Dobson-Stone C., Rubio J.P., Danek A., Chalmers R.M., Wood N.W. (2001). A conserved sorting-associated protein is mutant in chorea-acanthocytosis. Nat. Genet..

[78] Balikova I., Lehesjoki A.E., de Ravel T.J., Thienpont B., Chandler K.E., Clayton-Smith J. (2009). Deletions in the VPS13B (COH1) gene as a cause of Cohen syndrome. Hum. Mutat..

[79] Darvish H., Bravo P., Tafakhori A., Azcona L.J., Ranji-Burachaloo S., Johari A.H. (2018). Identification of a large homozygous VPS13C deletion in a patient with early-onset parkinsonism. Mov. Disord..

[80] Kistol D., Tsygankova P., Bostanova F., Orlova M., Zakharova E. (2024). New case of spinocerebellar ataxia, autosomal recessive 4, due to VPS13D variants. Int. J. Mol. Sci..

[81] Dziurdzik S.K., Conibear E. (2021). The Vps13 family of lipid transporters and its role at membrane contact sites. Int. J. Mol. Sci..

[82] Li P., Lees J.A., Lusk C.P., Reinisch K.M. (2020). Cryo-EM reconstruction of a VPS13 fragment reveals a long groove to channel lipids between membranes. J. Cell Biol..

[83] Kumar N., Leonzino M., Hancock-Cerutti W., Horenkamp F.A., Li P., Lees J.A. (2018). VPS13A and VPS13C are lipid transport proteins differentially localized at ER contact sites. J. Cell Biol..

[84] Guillen-Samander A., Leonzino M., Hanna M.G., Tang N., Shen H., De Camilli P. (2021). VPS13D bridges the ER to mitochondria and peroxisomes via miro. J. Cell Biol..

[85] Shen J.L., Fortier T.M., Zhao Y.G., Wang R., Burmeister M., Baehrecke E.H. (2021). Vmp1, Vps13D, and Marf/Mfn2 function in a conserved pathway to regulate mitochondria and ER contact in development and disease. Curr. Biol..

[86] Baldwin H.A., Wang C., Kanfer G., Shah H.V., Velayos-Baeza A., Dulovic-Mahlow M. (2021). VPS13D promotes peroxisome biogenesis. J. Cell Biol..

[87] McEwan D.G., Ryan K.M. (2022). ATG2 and VPS13 proteins: molecular highways transporting lipids to drive membrane expansion and organelle communication. FEBS J..

[88] Claude-Taupin A., Jia J., Bhujabal Z., Garfa-Traore M., Kumar S., da Silva G.P.D. (2021). ATG9A protects the plasma membrane from programmed and incidental permeabilization. Nat. Cell Biol..

[89] De Tito S., Almacellas E., Dai Yu D., Millard E., Zhang W., de Heus C. (2025). ATG9A and ARFIP2 cooperate to control PI4P levels for lysosomal repair. Dev. Cell.

[90] Peng K., Zhao G., Zhao H., Noda N.N., Zhang H. (2025). The autophagy protein ATG-9 regulates lysosome function and integrity. J. Cell Biol..

[91] Wu N., Cernysiov V., Davidson D., Song H., Tang J., Luo S. (2020). Critical role of lipid scramblase TMEM16F in phosphatidylserine exposure and repair of plasma membrane after pore formation. Cell Rep..

[92] Chakraborty S., Feng Z., Lee S., Alvarenga O.E., Panda A., Bruni R. (2024). Structure and function of the human apoptotic scramblase Xkr4. bioRxiv.

[93] Ji M., Li M., Sun L., Zhao H., Li Y., Zhou L. (2022). VMP1 and TMEM41B are essential for DMV formation during beta-coronavirus infection. J. Cell Biol..

[94] Hoffmann H.H., Schneider W.M., Rozen-Gagnon K., Miles L.A., Schuster F., Razooky B. (2021). TMEM41B is a pan-flavivirus host factor. Cell.

[95] Schneider W.M., Luna J.M., Hoffmann H.H., Sánchez-Rivera F.J., Leal A.A., Ashbrook A.W. (2021). Genome-scale identification of SARS-CoV-2 and pan-coronavirus host factor networks. Cell.

[96] Zhao Y.G., Chen Y., Miao G., Zhao H., Qu W., Li D. (2017). The ER-Localized transmembrane protein EPG-3/VMP1 regulates SERCA activity to control ER-Isolation membrane contacts for autophagosome formation. Mol. Cell.

[97] Jiang X., Fulte S., Deng F., Chen S., Xie Y., Chao X. (2022). Lack of VMP1 impairs hepatic lipoprotein secretion and promotes non-alcoholic steatohepatitis. J. Hepatol..

[98] Morita K., Hama Y., Mizushima N. (2019). TMEM41B functions with VMP1 in autophagosome formation. Autophagy.

[99] Li Y., Chao X., Yang L., Lu Q., Li T., Ding W.X. (2018). Impaired fasting-induced adaptive lipid droplet biogenesis in liver-specific Atg5-Deficient mouse liver is mediated by persistent nuclear factor-like 2 activation. Am. J. Pathol..

[100] Ding W.X., Ni H.M., Waguri S., Komatsu M. (2022). Lack of hepatic autophagy promotes severity of liver injury but not steatosis. J. Hepatol..

[101] Komatsu M., Waguri S., Koike M., Sou Y., Ueno T., Hara T. (2007). Homeostatic levels of p62 control cytoplasmic inclusion body formation in autophagy-deficient mice. Cell.

[102] Ni H.M., Woolbright B.L., Williams J., Copple B., Cui W., Luyendyk J.P. (2014). Nrf2 promotes the development of fibrosis and tumorigenesis in mice with defective hepatic autophagy. J. Hepatol..

[103] Chao X., Wang S., Fulte S., Ma X., Ahamed F., Cui W. (2022). Hepatocytic p62 suppresses ductular reaction and tumorigenesis in mouse livers with mTORC1 activation and defective autophagy. J. Hepatol..

[104] Wang X., Wang H., Xu B., Huang D., Nie C., Pu L. (2021). Receptor-mediated ER export of lipoproteins controls lipid homeostasis in mice and humans. Cell Metab..

[105] Wang Y., Liu L., Zhang H., Fan J., Zhang F., Yu M. (2016). Mea6 controls VLDL transport through the coordinated regulation of COPII assembly. Cell Res..

[106] Rong X., Wang B., Dunham M.M., Hedde P.N., Wong J.S., Gratton E. (2015). Lpcat3-dependent production of arachidonoyl phospholipids is a key determinant of triglyceride secretion. Elife.

[107] Hoffmann T.J., Theusch E., Haldar T., Ranatunga D.K., Jorgenson E., Medina M.W. (2018). A large electronic-health-record-based genome-wide study of serum lipids. Nat. Genet..

[108] Chu A.Y., Guilianini F., Grallert H., Dupuis J., Ballantyne C.M., Barratt B.J. (2012). Genome-wide association study evaluating lipoprotein-associated phospholipase A2 mass and activity at baseline and after rosuvastatin therapy. Circ. Cardiovasc. Genet..

[109] Sun L., Zhao C., Fu Z., Fu Y., Su Z., Li Y. (2021). Genome-scale CRISPR screen identifies TMEM41B as a multi-function host factor required for coronavirus replication. PLoS Pathog..

[110] Kratzel A., Kelly J.N., V'Kovski P., Portmann J., Bruggemann Y., Todt D. (2021). A genome-wide CRISPR screen identifies interactors of the autophagy pathway as conserved coronavirus targets. PLoS Biol..

